# Exploring the amphibian exposome in an agricultural landscape using telemetry and passive sampling

**DOI:** 10.1038/s41598-018-28132-3

**Published:** 2018-07-03

**Authors:** Jennifer E. Swanson, Erin Muths, Clay L. Pierce, Stephen J. Dinsmore, Mark W. Vandever, Michelle L. Hladik, Kelly L. Smalling

**Affiliations:** 1 Iowa State University, Department of Natural Resource Ecology and Management, Ames, IA 50011 USA; 2 US Geological Survey, Fort Collins Science Center, Fort Collins, CO 80526 USA; 3 US Geological Survey, Iowa Cooperative Fish and Wildlife Research Unit, Ames, IA 50011 USA; 4 US Geological Survey, California Water Science Center, Sacramento, CA 95819 USA; 5 US Geological Survey, New Jersey Water Science Center, Lawrenceville, NJ 08648 USA

## Abstract

This is the first field study of its kind to combine radio telemetry, passive samplers, and pesticide accumulation in tissues to characterize the amphibian exposome as it relates to pesticides. Understanding how habitat drives exposure in individuals (*i*.*e*., their exposome), and how that relates to individual health is critical to managing species in an agricultural landscape where pesticide exposure is likely. We followed 72 northern leopard frogs (*Lithobates pipiens*) in two agricultural wetlands for insight into where and when individuals are at high risk of pesticide exposure. Novel passive sampling devices (PSDs) were deployed at sites where telemetered frogs were located, then moved to subsequent locations as frogs were radio-tracked. Pesticide concentration in PSDs varied by habitat and was greatest in agricultural fields where frogs were rarely found. Pesticide concentrations in frogs were greatest in spring when frogs were occupying wetlands compared to late summer when frogs occupied terrestrial habitats. Our results indicate that habitat and time of year influence exposure and accumulation of pesticides in amphibians. Our study illustrates the feasibility of quantifying the amphibian exposome to interpret the role of habitat use in pesticide accumulation in frogs to better manage amphibians in agricultural landscapes.

## Introduction

Pesticides are one of multiple stressors that contribute to amphibian declines, but their population-level effects on health are still poorly understood^1,2^. Our knowledge of the impact of pesticides on amphibians is limited in part because route, timing, and rate of exposure in the field is unknown^3^. Pesticides occur frequently in amphibian habitats and accumulate in their tissues^4–7^; however, pesticide exposure relative to habitat use at the population or individual level has not been investigated. Sensitivity to pesticides varies by amphibian species and by the type of chemical or chemical mixtures to which an animal is exposed^8^. Timing of exposure is also important, and metabolism and excretion play a role in pesticide accumulation and residence time within tissues^8,9^. Compounding this complexity is the aquatic/terrestrial duality of many amphibian species. Due to their unique physiology and life history, amphibians are more vulnerable to pesticides than other wildlife species^10,11^. In addition, amphibians use both aquatic and terrestrial habitats, which are prone to change not only between years but also within a single season, contributing to fluctuations in pesticide presence and concentration^3,12^. Furthermore, amphibians have highly permeable skin which allows for dermal uptake of pesticides^13,14^. Contact with pesticides can cause death or a range of sub-lethal effects^2^. Health effects vary from immune suppression to reproductive and behavioral changes that may lead to population declines^15–18^. This is especially true in the agricultural landscape where pesticides are frequently applied but vary in type and amount^19,20^.

A better understanding of exposure potential will provide insight into threats that pesticides pose to amphibian health. Researchers have proposed that the terrestrial environment has the potential to significantly contribute to an amphibian’s pesticide accumulation and is a topic that warrants further investigation^13,17,21^. For example, some species (*e*.*g*., toads and wood frogs [*Lithobates sylvaticus*]) spend much of their adult life in terrestrial environments, and may be exposed to greater amounts of pesticides outside of wetland habitats^21,22^; however, this has not been tested in the field. Several field studies have noted that pesticides detected in frog tissues could not be explained by the aquatic habitats in which they were captured^5–7^, leaving questions about the role of habitat use in pesticide exposure and accumulation. To our knowledge, pesticide exposure based on habitat use has never been studied explicitly in any amphibian species under field conditions.

Understanding the complex relationships among amphibians, pesticides, and their environments is vital for conservation, and investigating pesticide exposure in the field is a central piece of this understanding. Specifically, to design mitigation strategies, we need to know where and when individuals are at the highest risk of pesticide exposure. Our goal was to understand the role of habitat use in determining pesticide exposure in frogs in an agricultural landscape. This information will facilitate an assessment of the potential health risks of agrochemicals under field conditions. We hypothesized that habitat use, measured as time spent in a particular habitat, would influence an individual frog’s exposure to types and amounts of pesticides, which would correlate with the types of pesticides accumulating in their tissue.

**Figure 1:**
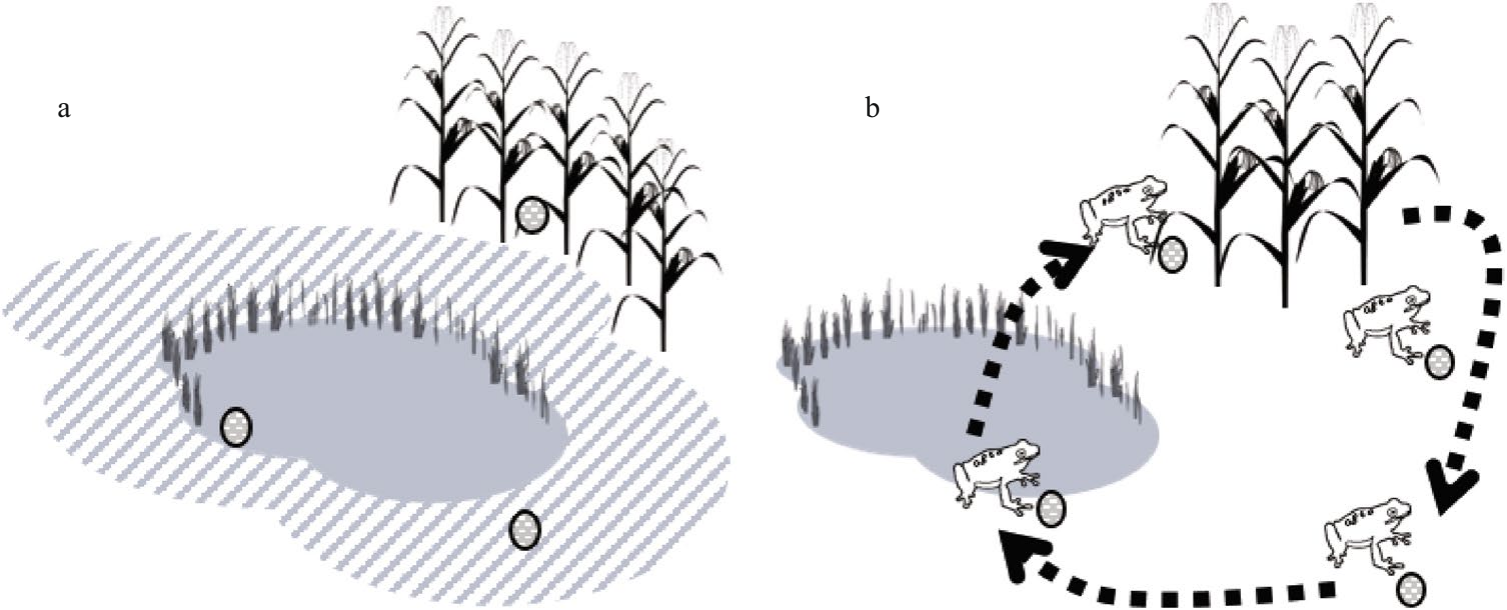
Study design. (**a**) PSDs deployed in three habitat types (wetland, grassland, and agriculture) at both sites from May–August, 2015–2016. PSDs were placed in a single location for 2 wks, collected for analysis and replaced. Ovals = PSD, grey shaded area = wetland habitat, grey striped area = grassland buffer habitat, and corn stalks = agricultural habitat. (**b**) Each deployed PSD was also associated with an individual telemetered frog (n = 9). Each PSD was moved to mirror the frog’s locations as it moved among different habitat types (dashed arrow). PSDs were moved with the frog for 2 wks, collected for analysis and replaced with another PSD.

To our knowledge, this is the first field study to combine radio telemetry, passive samplers, and pesticide accumulation in tissues as a means to characterize the amphibian exposome as it relates to pesticides. We used a novel application of a silicone Passive Sampling Device (PSD)^23^ to assess pesticide presence near individual animals occupying different habitat types, and to facilitate comparisons of pesticides in habitats to pesticides in tissues of northern leopard frogs (*Lithobates pipiens*; Fig. 1). We also used radio telemetry to assess survival and determine the amount of time individual frogs spent in particular habitats relative to pesticide exposure. We compared pesticide type and concentration among different habitats and in frog tissue to determine if a relationship exists between a frog’s habitat use and their pesticide accumulation.

## Results

### Radio telemetry

We radio tracked 72 northern leopard frogs during the summers of 2015 and 2016 (M = 31, F = 41; see Supplementary Tables S1 and S2). We tracked an equal number of frogs at two constructed agricultural wetlands in Cerro Gordo and Worth Counties, IA, USA. Both sites were surrounded by a grassed buffer but were in close proximity (20–750 m) to corn and soybean fields on all sides (see Supplementary Fig. S1). Individuals were tracked for an average of 29 days (minimum = 3, maximum = 84). During the study, 20 frogs died (28%), 24 frogs could not be relocated (33%), and 28 frogs were captured and euthanized for tissue analysis (39%).

**Figure 2:**
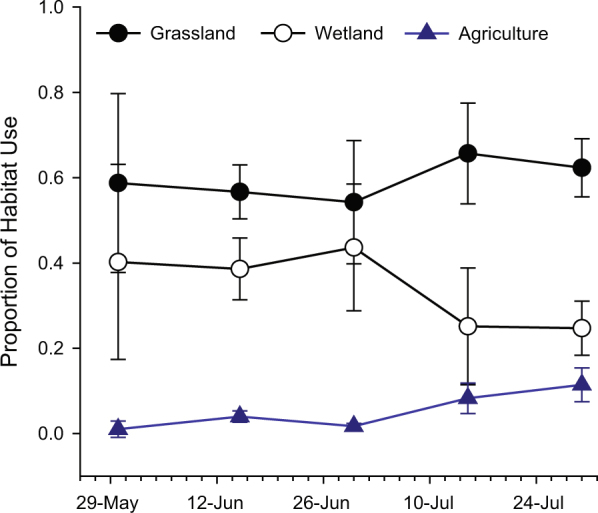
Change in the proportion of habitat used by radio tracked northern leopard frogs (n = 72) in north-central Iowa, May – August in 2015 and 2016 with associated Standard Error (SE).

We analyzed the home ranges of individuals with ≥20 recorded locations (F = 23, M = 18). Females had a larger home range than males, but total movement and average daily movement for male frogs were greater than for female frogs (Table 1). Fifty-eight percent of frog locations were in grassland, 34% in wetland, and 6% in agricultural fields (but only 7 of 72 individuals spent three or more consecutive days in agricultural fields). Frogs selected for grassland and wetland habitats (3.89 ± 0.29 Global Selection Ratio [GSR] and 3.57 ± 0.43 GSR, respectively), and avoided agriculture, forest, and developed habitats (0.09 ± 0.02; 0.17 ± 0.08; 0.03 ± 0.02 GSRs, respectively). Use of habitat by frogs changed over time (Fig. 2). Northern leopard frogs primarily used a mixture of grasslands and wetlands from mid-May until late June, then wetland habitat use declined in July and continued to decline through the summer. There was an increase in the use of grassland and agricultural habitat beginning in July.

**Table 1:** Median home ranges (m^2^) and distances moved (m) with minimum and maximum values for female and male radio telemetered northern leopard frogs in Iowa 2015–2016.

	Core Home Range		Total Home Range		Total Movement		Daily Movement	
	Median	Range	Median	Range	Median	Range	Median	Range
Females	1,347	240 to 64,157	6,423	981 to 246,661	389	2 to 1,680	16	1 to 41
Males	3,154	239 to 11,800	4,740	2,640 to 70,017	553	7 to 1,405	18	2 to 59

Core home range = 50% quantile and total home range = 95% quantile. Total movement represents the total distance a frog moved while it was tracked and daily movement is the average distance a frog moved per day while it was tracked (total movement/number of days tracked).

Five of our 12 models (ΔAICc < 2; Table 2) were at least partially supported by the data. The model with the most support indicated that survival varied by sex and habitat type used, specifically that survival probability is lower in females, lowest in grassland habitats and highest in agricultural habitats. Survival estimates (both sexes) for the length of our study (3 mo) were extrapolated from estimated daily values by multiplying by 3 months. Three-month survival probabilities were highest in agriculture (0.62 ± 0.10), intermediate in wetlands (0.46 ± 0.92), and lowest in grasslands (0.28 ± 0.07).

**Table 2:** Results of known fate models for northern leopard frogs in Iowa, 2015–2016 with support (ΔAICc < 2).

Model Name	ΔAICc	*w*	k	Deviance
S(Sex + Habitat)	0.00	0.16	3	211.45
S(+Habitat)	0.43	0.13	2	213.89
S(Year + Habitat)	0.55	0.12	3	201.12
S(Group + Habitat)	1.76	0.06	9	213.41
S(Site + Habitat)	1.96	0.06	3	221.01

AICc of the top model S(Sex + Habitat) was 217.50, *w* = AICc Weight, and k = number of parameters.

### Passive Sampling Devices (PSDs)

We analyzed PSDs that were associated with telemetered frogs (n = 9) as they moved across the landscape for over 100 pesticides and pesticide degradates (Fig. 1). Twelve different pesticides were detected, and the number of pesticides to which individual frogs were potentially exposed during the time they were tracked ranged from 9–12 (median = 12; 6 fungicides, 2 herbicides and 4 insecticides; see Supplementary Table S3). The three pesticides detected at the highest concentrations in PSDs were chlorpyrifos (organophosphate insecticide), pyraclostrobin (fungicide), and azoxystrobin (fungicide).

**Figure 3:**
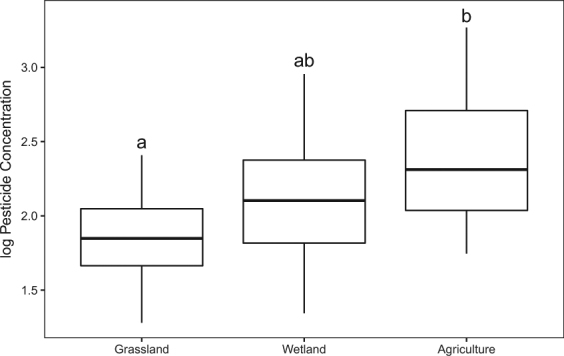
Log normalized concentrations of pesticides detected in environmental PSDs (ng/PSD) placed in different habitat types in Iowa, 2015–2016. Letters above boxplots indicate differences (P < 0.05) among habitat types (see Supplementary Table S4 for list of pesticides detected).

Seventeen pesticides (8 fungicides, 6 insecticides, and 3 herbicides; see Supplementary Table S4) were observed in PSDs placed in habitats in 2015 and 2016 (single location, not moved with telemetered frogs Fig. 1a). The three pesticides detected at the highest concentrations in the PSDs were chlorpyrifos, metolachlor (herbicide), and azoxystrobin (fungicide). The highest concentration of pesticides was observed in agricultural habitats, followed by wetland and then grassland habitats (Table 3). The concentrations of pesticides detected in PSDs in grassland habitats were lower than agricultural habitats (P < 0.01) and wetlands were not different than either grassland or agricultural habitats (P = 0.06 and 0.05, respectively; Fig. 3). There were no significant differences between the total numbers of pesticides observed among habitat types.

**Table 3:** Median number and concentration of pesticides (ng/PSD) found in silicone PSDs placed in grassland, wetland, and agricultural habitats in Iowa, USA 2015 and 2016 (see Supplementary Table S4 for list of pesticides detected).

Habitat	Number of Pesticides: Median	Number of Pesticides: Range	Pesticide Concentration: Median	Pesticide Concentration: Range
Grassland	4	2 to 11	60	7 to 250
Wetland	6	2 to 11	122	10 to 896
Agriculture	6	2 to 11	197	46 to 1,844

Concentrations of pesticides did not vary over time within a particular habitat, based on data from the PSDs placed independent of frogs and collected at 2 wk intervals for 3 mos during the summer (see Supplementary Table S4). The number of pesticides in agricultural fields also did not differ from May to August, but the number of pesticides in wetlands and in grassland habitats varied over time. In both cases the number of pesticides detected peaked in July and was higher (P < 0.01) than the number detected earlier in the spring or later in the summer.

In the winter samples (PSDs placed in aquatic locations; see Supplementary Fig. S1), the herbicides atrazine and metolachlor, and the insecticide chlorpyrifos, were detected at both the Cerro Gordo (20.2, 16.6, and 2.6 ng/PSD, respectively) and Worth wetlands (16.0, 17.5, and 0.42 ng/PSD, respectively). The pyrethroid insecticide bifenthrin was detected at the Worth wetland only (0.50 ng/PSD).

### Frog tissues

**Figure 4:**
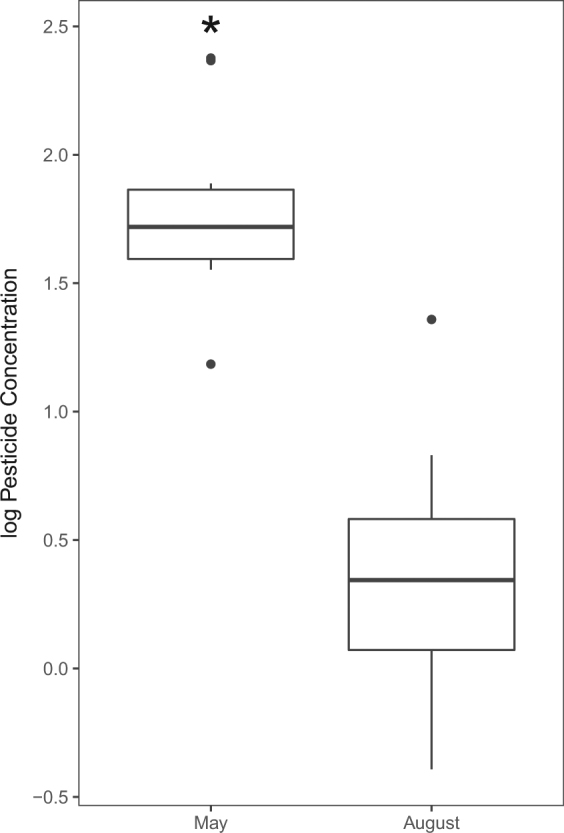
Log normalized concentrations of pesticides detected in frog gonad and liver tissues (ng/g) May vs August, 2016 in Iowa, USA. Asterisk (*) indicates difference (P < 0.05; see Supplementary Table S5 for list of pesticides detected).

The number of pesticides in frog livers and gonads collected in 2016 ranged from 0–3 (median = 3) and included 2 fungicides (fenbuconazole and tebuconazole), 1 insecticide (bifenthrin), and 1 breakdown product of DDT (*p*, *p*’-DDE; Table 4, see Supplementary Table S5). Frogs were collected in mid-May and early August in 2016 to characterize potential differences in pesticide accumulation as frogs moved from the wetland to the terrestrial environment. Both the number of pesticides detected in the livers and gonads and their respective concentrations were higher in frogs collected in May compared to those collected in August (P < 0.01 and < 0.01, respectively; Fig. 4). *p*, *p*’- DDE was not included in further analyses of the tissue data, because it is persistent in the environment and would not degrade within the time frame of our study^24^.

**Table 4:** Total pesticide concentrations (sum of all pesticide detected) and the number of pesticides in the liver and gonad tissue of telemetered northern leopard frogs vs their associated PSDs that “followed” them over time and the proportion of time the frogs and their PSDs spent in each habitat type (Fig. 1b).

Site	Frog ID	Concentration of Pesticides		Number of Pesticides		Proportion of habitat (%)			
		Frog Tissue (ng/g)	PSDs (ng/PSD)	Frog Tissue (ng/g)	PSDs (ng/PSD)	Grass	Wetland	Agriculture	Other
CG	60.3	20.1	86.3	1	9	0.82	0.18	0.00	0.00
CG	89.2	9.6	73.6	3	9	0.68	0.00	0.21	0.11
W	26.3	6.65	159	2	12	0.84	0.16	0.00	0.00
W	18	ND	148	ND	12	0.85	0.06	0.00	0.09
W	4.4	ND	160	ND	12	0.84	0.16	0.00	0.00
W	13.2	ND	123	ND	10	0.11	0.17	0.72	0.00
W	42	21.1	147	1	12	0.04	0.78	0.00	0.00
W	45	ND	147	ND	12	0.84	0.14	0.02	0.00
W	48	ND	69.3	ND	10	0.65	0.00	0.35	0.00

Frog ID = Individual frog identification number. Pesticide concentrations are in ng/g for frog tissue and ng/PSD for PSDs, Site: CG = Cerro Gordo and W = Worth, “ND” = Not detected.

## Discussion

Information from this study using a combination of radio telemetry and PSDs to document exposure and accumulation in tissues expands our understanding of the amphibian exposome as it relates to pesticides. Our study is likely the first to assess questions of pesticide exposure and habitat selection on adult animals based on field data, and supports the importance of the terrestrial environment and the notion that amphibians are at risk of exposure in terrestrial habitats^13,21^. Data showing higher tissue pesticide concentration detected in May relative to August suggest that the greatest vulnerability of adult frogs to pesticide accumulation, in the agricultural landscape, may be during winter hibernation and spring breeding when their use of wetlands is most intense (Fig. 2). Although, transient exposures in agricultural fields are likely to be of long-term importance and should not be discounted despite the low percentage of time that frogs spend there. Pesticide research with amphibians typically focuses on larval stages, but a reduction in adult (and juvenile) survival rates is more likely to presage population decline^25^, suggesting that our information on adults is timely.

Results supported our expectation of an influence of habitat use on survival rates. However, we suggest that this was because of increased predation in grassland habitats rather than differences in pesticide exposure. Pesticide exposure in the field is generally sublethal for amphibians^26,27^ including leopard frogs^26^, and it is unlikely that we would have captured sublethal effects on survival in our 3 mo survival probabilities. Pesticides observed in amphibian habitats with documented sublethal effects include herbicides, atrazine and metalochlor, the organophosphate insecticide, chlopyrifos, and several fungicides. Atrazine is responsible for a variety of estrogenic effects, reduced immune response, and behavior modification^28^. Metolachlor slows development and growth and has negative effects on the thymus gland^27,29^, and synergistically impacts the effects of atrazine^27,28^. Chlorpyrifos causes increased time to metamorphosis^30^ while exposure to fungicide formulations containing pyraclostrobin decreases growth and development in larval amphibians^31^.

Frogs in our study spent most of their time (58%) in grasslands, the habitat where pesticide exposure was the lowest, but where predation risk to amphibians is typically higher than in wetlands^32,33^. Additionally, the highest percentage of radio-tracked frogs that died of predation were found in grassland habitats (47%). Thus, for this short-term study, it is likely that differences in survival relative to habitat are indicative of predation risk rather than exposure to pesticides. However, in terms of management, frogs did spend a small but measurable amount of time in agricultural fields (on average frogs spent 6% of their time in a corn or soybean field) and because the effects of pesticides are accumulative^2,27^, even short periods of time spent in habitats where pesticide exposure is high can have negative consequences over their lifetime, especially when use of agricultural fields coincides with pesticide application^2,34^.

Northern leopard frogs, like many ranid species, hibernate at the bottom of lakes and ponds, resting on the substrate and covered with a thin layer of silt that allows for cutaneous uptake of O_2_. Understanding potential exposure during hibernation is another piece of the exposome that is understudied. Amphibian skin is highly permeable, a characteristic that is magnified during hibernation, when frogs undergo physiological changes at low temperatures (<5 °C). These changes lower metabolism which reduces the metabolism of pesticides^35–37^. Liver condition and function is also lowest at the end of hibernation (early spring), which makes it more difficult for an individual to efficiently metabolize contaminants^38^. In our study, four pesticides (two herbicides, atrazine and metolachlor, and two insecticides, bifenthrin and chlorpyrifos) were identified in overwintered (aquatic) PSDs, with atrazine and metolachlor at the highest concentrations. These results indicate that exposure during winter hibernation is significant for northern leopard frogs.

In the spring, northern leopard frogs selected for aquatic habitats coincident with the application of herbicides (May, June) and then moved into terrestrial habitats (July, August) when insecticides are commonly applied (*i*.*e*., chlorpyrifos peaked in August in all habitat types). Frogs used agricultural fields infrequently indicating that the pesticide application rates (exposure concentrations) tested in laboratory studies^21^ may not represent the actual environmental concentrations to which frogs are exposed in their preferred grassland habitats.

Pesticide accumulation in tissues was assessed in telemetered frogs in May that were spending a high proportion of their time in aquatic habitats and again in August in frogs spending a high proportion of their time in terrestrial environments (grasslands and agricultural fields; Fig. 2). Higher pesticide concentrations were observed in frog tissue collected in May compared to August. Other studies have reported the accumulation of similar types and concentrations of pesticides in frog tissues (liver and whole bodies) collected from wetland habitats early in the summer^5–7^, however, no information on pesticide exposure and resulting accumulation in frogs occupying more terrestrial habitats is available for comparison. Anurans have been shown to exhibit reduced dermal uptake of pesticides from soil while dehydrated^39^, a potential contributing factor in our study where pesticide concentration in leopard frog tissue was lowest while they were occupying terrestrial habitats later in the summer.

A number of pesticides were identified in PSDs (representing potential exposure) that were not detected in frog tissues. Other studies have reported differences in pesticides identified in the environment (water and sediment) compared to those accumulating in tissue^5–7^. These differences can be attributed to several factors, (1) the physical-chemical properties of the pesticides (may not be accumulated), (2) metabolism/elimination by the frog (accumulated but quickly broken down), or (3) limited time spent in each habitat (slow assimilation into tissues). In contrast, two pesticides (fenbuconazole and *p*, *p*’-DDE) were only identified in frog tissues. Discrepancies between pesticides identified in tissues versus the environment could be related to low environmental concentrations, limitations in the ability of PSDs to sequester more hydrophobic compounds – although this is unlikely^23,40^, or effect of maternal transfer rather than dermal uptake.

Our results corroborate the importance of multiple habitat types to anurans, highlighting the need to protect wetlands and surrounding habitats (*e*.*g*., grass buffers). We assessed pesticide exposure in the environment and differences in the use of habitat by frogs, relative to pesticides found in their tissues, to understand how these factors interact to affect the persistence of frogs on the landscape. These data illustrate that understanding the extent and magnitude of pesticide exposure is foundational in assessing risk and developing mitigation strategies for the conservation of frogs; and our study was unique in its innovative use of silicone PSDs to facilitate these assessments. More broadly, the distances frogs moved, patterns of habitat use, and home range sizes are similar to previous telemetry studies on northern leopard frogs in the Midwestern United States^41,42^, such that our findings may be applicable to other altered (*i*.*e*., agricultural) landscapes. This study provides initial steps towards quantifying the amphibian exposome and interpreting the role of habitat use in pesticide accumulation as it contributes to population declines.

## Methods

### Study Sites and Species

This study was conducted in northern Iowa, USA at two constructed wetlands (see Supplementary Fig. S2). Wetlands were embedded in a highly agricultural landscape that consisted mainly of annual row crops (corn and soybeans). Each wetland had an associated grass buffer (buffer:wetland ratio = 4:1), but were surrounded on all sides by agricultural fields (ranging from 20–750 m away; see Supplementary Table S6). Wetlands were built to catch and hold subsurface tile drainage from surrounding agricultural fields to remove nitrate from local surface water bodies^43^.

We chose northern leopard frogs as our study species because they are (1) appropriately sized for radio telemetry, (2) relatively abundant in our study area, and (3) similar to other anuran species in their use of both aquatic and terrestrial habitat. Northern leopard frogs are distributed widely across the United States and parts of Canada^44^. Although considered a fairly common species, declines or extinctions have been observed in parts of their range^45,46^. Northern leopard frogs overwinter by resting on the substrate in waterbodies that do not freeze completely^47^. Breeding and egg deposition occur in aquatic environments during the spring^48^. Post-breeding, adults move to grassy, terrestrial habitats for feeding before returning to waterbodies for hibernation in the fall^44,49^.

### Radio telemetry

We used radio telemetry to assess survival probability, home range, distance moved, and habitat types used by frogs in 2015 and 2016. In May we captured 10 adult frogs at each study site (see Supplementary Table S2). Frogs appeared healthy (no observable disease) and were taken to a field laboratory and implanted with an internal radio transmitter (17 × 8.5 × 5.5 mm, 1.8 g; BD-2H in 2015 and 27 × 8.5 × 5.5 mm, 1.8g; BD-2HX in 2016, Holohil Systems Ltd., Canada)^50^. Frogs were located daily, from May release, until August. We recorded the habitat type where frogs were observed and marked their location with GPS (Garmin eTrex® 30). Frogs were recorded in 1 of 5 habitats: grassland (grass buffers, pastures, or prairies); wetland (wetlands, streams and other small water bodies); agriculture (corn and soybean fields); forest (large groups of deciduous trees); or developed (man-made structures such as roads). If individuals died or could not be located for >2 wks they were replaced with newly captured and telemetered individuals (see Supplementary Table S2). This study was approved by the Iowa State University Institutional Animal Care and Use Committee (protocol #3-15-7989-D) and performed in accordance with protocol guidelines.

### Passive Sampling Device (PSD) deployment and analysis

We used a novel application of a silicone PSD technique^23^ to quickly and easily screen for potential pesticide exposure. Commercially available silicone wristbands were purchased (width: 2.5 cm; inner diameter 6.7 cm; 24hourwristbands.com, Houston, TX) and cleaned^23^. Three PSDs were set aside as “cleaning blanks”, frozen, and extracted with the environmental samples. Additionally, during each PSD field deployment a “field blank” was also deployed at the site to assess potential contamination. Clean latex gloves were worn when handling PSDs. In 2016, we assigned each telemetered frog (initial n = 10/site) a PSD (Fig. 1b). That PSD was secured to the substrate at the frog’s initial location, then relocated each time the frog moved (see Supplementary Fig. S3). Because individual frogs were relocated daily, PSDs were deployed at a particular location no more than 24 hrs longer than a frog. Thus, the PSD “experienced” the same habitat encountered by that frog and provided daily information on an individual’s exposure to pesticides as it moved across the landscape. PSDs were moved with each frog for 2 wks then collected and replaced throughout the tracking period 2–4 times depending on how long the frog was tracked.

We also used PSDs to assess the potential pesticide exposure by placing PSDs (independent of frogs) in habitats where we most frequently observed our telemetered animals (Fig. 1a). We deployed PSDs in four locations at each site in a combination of grassland, wetland, and agricultural habitats (*e*.*g*., two in grasslands, one in wetland, and one in agriculture), in July–August in 2015, and June–August in 2016 (see Supplementary Fig. S1). PSDs were anchored to the substrate using sterilized landscape staples. They were left in the environment for 2 wks and then collected and replaced with new PSDs in the same location.

Finally, we assessed the potential pesticide exposure in overwintering habitat by placing PSDs at four aquatic locations in each wetland from November 2015 to April 2016. PSDs were zip-tied to a metal O-ring that was attached to a metal T-post driven into the substrate in 1 m of water (see Supplementary Fig. S4). The PSDs were submerged and in contact with the substrate as a northern leopard frog would be during hibernation^51,52^. PSDs were not collected and replaced during the winter because sites were inaccessible due to ice cover.

All PSDs were sent to the USGS California Water Science Center for analysis. PSDs were extracted and analyzed for over 100 pesticides and pesticide degradates. In the laboratory, PSDs were rinsed gently with deionized water to remove particulates and allowed to dry in the fume hood. Prior to extraction PSDs were weighed and placed in a pre-cleaned flask. Samples were spiked with d_14_-trifluralin-, ring ^13^C_4_-fipronil, ring-^13^C_12_-*p*, *p*’-DDE and phenoxy-^13^C_6_-*cis*-permethrin as recovery surrogates, extracted twice with 50 mL of ethyl acetate using sonication for 2 hrs. Each extract was reduced to 1 mL, combined, reduced to 200 µL and analyzed for pesticides and pesticide degradates on an Agilent 7890 GC coupled to an Agilent 7000 MS/MS operating in electron ionization (EI) mode or an Agilent 1260 bio-inert LC coupled to an Agilent 6430 MS/MS. Data was collected in multiple reaction monitoring (MRM) mode with each compound having 1 quantifier MRM and at least 1 qualifier MRM. Limits of detection (LODs) for all compounds ranged from 2–5 ng/PSD.

### Frog tissue collection and analysis

In 2016 we captured and euthanized individuals in mid-May prior to tracking while they were breeding and residing at the wetlands (n = 10). In August we captured and euthanized all remaining radio telemetered frogs (n = 10). Frogs were euthanized with an overdose of Tricaine Methanesulfonate (0.5 g/1 L water)^53^. Individual liver and gonads were dissected in the field, frozen, and sent to the USGS California Water Science Center for analysis^6^.

All tissues were extracted using pressurized fluid extraction and analyzed for 98 pesticides and pesticide degradates using an Agilent 7890 GC coupled to an Agilent 7000 MS/MS operating in EI mode. Method detection limits (MDLs) for all compounds ranged from 0.5 to 4.2 μg/kg wet weight^5^ (see Supplementary Methods).

### Statistical analysis

We performed a known fate analysis in Program MARK (V6.1)^54^ to estimate survival probabilities of the radio tracked frogs (n = 72). We included daily habitat use (*i*.*e*., the habitat type of each location for individual frogs) as an individual covariate to evaluate its influence on frog survival. Each frog was assigned to 1 of 8 groups that reflected site, year, and sex (see Supplementary Table S7). Twelve models were fit to the data to explore survival among groups and habitat types (see Supplementary Table S8). We considered models where survival was constant or differed between site, sex, year, or among groups. We also fit models to include a daily habitat covariate using our classification of the five habitat types. Survival was not estimated in forest or developed habitats because of low use (Forest = 0.008% and Developed = 0.003% of recorded locations). Model selection was based on Akaike’s Information Criterion for small sample sizes (AIC_c_)^55^.

We estimated core home range (50% quantile) and total home range (95% quantile) of 41 frogs (18 M, 23 F; see Supplementary Table S2) that had ≥20 recorded GPS locations^56^. We used the “kde” and “isopleth” functions in Geospatial Modeling Environment (GME; V0.7.4.0)^57^ using kernel density estimates.

We estimated the daily distances (m) each frog moved using the “pointdistances” function in GME, which assumes a straight-line moved between successive locations. We summed the daily distances each frog moved and divided the total by the number of days the frog was tracked to get average daily movement.

We determined the proportional use of habitat types, relative to their availability by calculating GSRs using the Manly Resource Selection function in R (V3.3.3)^58^. Available habitat was calculated within a 1 km buffer^41,42^. GSRs reflect the proportion of time an animal uses each habitat relative to the proportion of that available habitat. For example, a habitat with a GSR > 1 indicates that the animal shows positive selection for that habitat.

We combined PSD data from 2015 and 2016 and used Analysis of Variances (ANOVAs) in R to test for a difference in number of pesticides and total pesticide concentration in the PSDs among the habitat types (whether PSD was placed in an agricultural, wetland, or grassland habitat). We also performed ANOVAs to test for a difference in number of pesticides and pesticide concentrations within habitat types over time (PSDs placed in early, mid, or late summer). Data were log transformed to meet assumptions of constant variance and normality. All non-detect values received ½ MDL for statistical analyses.

We assessed differences in pesticide presence in frog gonad and liver tissue using a Kruskal-Wallis test^59^ and an independent t-test to test for differences in concentration of pesticides found in individuals collected in May vs August 2016. Data were log transformed to meet assumptions of constant variance and normality. All non-detect values received ½ MDL for statistical analyses.

### Data availability

Data generated in this study are available at the U.S. Geological Survey data release, 10.5066/F7D799PV.

## Electronic supplementary material

Supplementary Information
