# Supplementary material for: Exploring the amphibian exposome in an agricultural landscape using telemetry and passive sampling
Source: Sci Rep. 2018 Jul 3;8:10045. doi: 10.1038/s41598-018-28132-3 (PMC6030078; doi:10.1038/s41598-018-28132-3)
Supplement: Supplementary file 1 — Supplementary Information [file 41598_2018_28132_MOESM1_ESM.pdf]

# Supplementary information: Exploring the amphibian exposome in an agricultural landscape using telemetry and passive sampling

**Jennifer E. Swanson[1,*], Erin Muths[2], Clay L. Pierce[3], Stephen J. Dinsmore[1], Mark W. Vandever[2], Michelle L. Hladik[4], and Kelly L. Smalling[5]**

[1] Iowa State University, Department of Natural Resource Ecology and Management, Ames, IA 50011, USA
[2] US Geological Survey, Fort Collins Science Center, Fort Collins, CO 80526, USA
[3] US Geological Survey, Iowa Cooperative Fish and Wildlife Research Unit, Ames, IA 50011, USA
[4] US Geological Survey, California Water Science Center, Sacramento, CA 95819, USA
[5] US Geological Survey, New Jersey Water Science Center, Lawrenceville, NJ 08648, USA
[*]jeswanson1@gmail.com

## Supplementary Methods

**Frog tissue analysis.** Tissue samples were thawed and homogenized with $Na_2SO_4$ using a clean, solvent-rinsed mortar and pestle. Samples were spiked with $d_{14}$trifluralin, ring-$^{13}C_{12}$-*p,p′*-DDE and phenoxy-$^{13}C_6$-*cis*-permethrin as recovery surrogates and extracted with dichloromethane (DCM) using pressurized liquid extraction. Sample extracts were then dried over $Na_2SO_4$, reduced and 10% by volume of each raw extract was allowed to evaporate to a constant mass for gravimetric lipid determination. A majority of the lipid was removed using gel permeation chromatography followed by 6% deactivated Florisil previously activated at 550 °C for 16 h. Prior to analysis, samples were reduced to 0.2 mL, and a deuterated internal standard was added to each extract. Sample extracts were analyzed on an Agilent 7890 GC coupled to an Agilent 7000 MS/MS operating in EI mode. Data was collected in multiple reaction monitoring (MRM) mode with each compound having 1 quantifier MRM and at least 1 qualifier MRM. Method detection limits (MDLs) for all compounds ranged from 0.5 to 4.2 μg/kg wet weight (Smalling et al. 2013).

**Pesticide quality control measures**. All sample glassware was hand-washed and rinsed with tap water followed by acetone and hexane prior to use. All solvents and other reagents were American Chemical Society (ACS) grade or better Pesticide standard materials were donated by the USEPA National Pesticide Repository. Purities ranged from 95 to 99%. Performance-based quality assurance and quality control included the parallel analysis of procedural blanks, matrix spikes, and replicates in 10% of the samples analyzed. PSD cleaning and field blanks did not contain detectable levels of pesticides. PSD cleaning and field blanks as well as laboratory procedural blanks run with each batch of tissue samples did not contain detectable levels of pesticides. Means (± standard deviation) of $d_{14}$-trifluralin, ring $^{13}C_4$-fipronil, ring-$^{13}C_{12}$-*p,p′*-DDE and phenoxy-$^{13}C_6$-cis-permethrin added prior to the extraction of the PSDs as recovery surrogates were 90 ± 11%, 100 ± 13%, 99 ± 9%and 97 ± 6%, respectively. Means (± standard deviation) of t $d_{14}$trifluralin, ring-$^{13}C_{12}$-*p,p′*-DDE and phenoxy-$^{13}C_6$-*cis*-permethrin added prior to tissue sample extraction as recovery surrogates were 93 ± 16%, 93 ± 15%, and 95 ± 15%, respectively. Matrix spikes were analyzed in 10% tissue samples and the recoveries ranged from 66% to 129% (median of 102%), 70% – 126% (median of 99%) and 65% to 108% (median of 84%), respectively. PSDs and tissue for pesticide analysis were stored frozen at −20 °C and held for no longer than 6 mo prior to extraction.

| Site | Year | Females | Males |
|---|---|---|---|
| Cerro Gordo | 2015 | 11 | 8 |
| Worth | 2015 | 8 | 11 |
| Cerro Gordo | 2016 | 8 | 9 |
| Worth | 2016 | 14 | 3 |

**Table S1**. Numbers and sexes of 72 radio tracked northern leopard frogs in Cerro Gordo and Worth counties, Iowa, USA 2015-2016.

| ID # | Site | Year | Sex | Mass (g) | Start Date | End Date | # Locations | Proportion of locations | | | | | Home Range | Fate |
|---|---|---|---|---|---|---|---|---|---|---|---|---|---|---|
| | | | | | | | | W | G | A | F | D | | |
| 0 | CG | ‘15 | M | 38 | 6/3 | 6/25 | 20 | 40 | 50 | 10 | 0 | 0 | 28,316 | Lost |
| 1 | W | ‘15 | F | 24 | 6/24 | 7/9 | 15 | 40 | 60 | 0 | 0 | 0 | - | Dead |
| 4 | CG | ‘15 | M | 20 | 6/24 | 8/10 | 31 | 35 | 39 | 26 | 0 | 0 | 45,783 | Captured |
| 6 | W | ‘15 | F | 24 | 6/10 | 7/5 | 22 | 9 | 91 | 0 | 0 | 0 | 2,445 | Dead |
| 6.2 | CG | ‘15 | M | ND | 7/10 | 7/16 | 7 | 14 | 71 | 14 | 0 | 0 | - | Lost |
| 13 | CG | ‘15 | M | 47 | 6/3 | 7/23 | 19 | 0 | 95 | 5 | 0 | 0 | - | Lost |
| 15 | W | ‘15 | M | 22 | 5/18 | 6/23 | 35 | 31 | 69 | 0 | 0 | 0 | 15,011 | Captured |
| 15.2 | W | ‘15 | M | 37 | 7/5 | 8/10 | 35 | 6 | 89 | 6 | 0 | 0 | 11,743 | Captured |
| 23 | W | ‘15 | M | 22 | 5/18 | 6/23 | 20 | 15 | 70 | 5 | 10 | 0 | 3,053 | Lost |
| 26 | W | ‘15 | M | 24 | 5/18 | 6/22 | 30 | 70 | 30 | 0 | 0 | 0 | 3,062 | Captured |
| 26.2 | CG | ‘15 | F | ND | 7/10 | 8/10 | 28 | 4 | 96 | 0 | 0 | 0 | 4,225 | Captured |
| 29 | CG | ‘15 | F | 36 | 5/18 | 5/28 | 9 | 33 | 67 | 0 | 0 | 0 | - | Dead |
| 29.2 | CG | ‘15 | M | 24 | 6/24 | 7/14 | 19 | 16 | 84 | 0 | 0 | 0 | - | Lost |
| 31 | CG | ‘15 | F | 43 | 5/18 | 6/24 | 29 | 34 | 52 | 14 | 0 | 0 | 112,648 | Captured |
| 31.2 | W | ‘15 | F | 42 | 7/5 | 8/10 | 34 | 9 | 76 | 6 | 9 | 0 | 15,957 | Captured |
| 34 | CG | ‘15 | F | 45 | 5/18 | 6/30 | 35 | 17 | 77 | 6 | 0 | 0 | 7,279 | Captured |
| 34.2 | CG | ‘15 | F | 21 | 7/10 | 8/8 | 20 | 5 | 80 | 15 | 0 | 0 | 26,492 | Lost |
| 40 | W | ‘15 | M | 23 | 5/18 | 6/6 | 15 | 33 | 67 | 0 | 0 | 0 | - | Lost |

| | | | | | | | | | | | | | | |
|---|---|---|---|---|---|---|---|---|---|---|---|---|---|---|
| 46 | W | ‘15 | M | 22 | 5/18 | 6/23 | 34 | 53 | 47 | 0 | 0 | 0 | 17,168 | Captured |
| 46.2 | CG | ‘15 | F | 21 | 7/5 | 8/11 | 20 | 50 | 45 | 5 | 0 | 0 | 11,905 | Captured |
| 49 | W | ‘15 | M | 22 | 5/18 | 6/14 | 26 | 19 | 77 | 4 | 0 | 0 | 13,961 | Lost |
| 53 | CG | ‘15 | M | 41 | 5/18 | 6/6 | 16 | 38 | 56 | 6 | 0 | 0 | - | Lost |
| 56 | W | ‘15 | M | 22 | 5/18 | 8/11 | 20 | 30 | 70 | 0 | 0 | 0 | 26,696 | Captured |
| 59 | W | ‘15 | F | 25 | 5/18 | 5/28 | 11 | 64 | 36 | 0 | 0 | 0 | - | Lost |
| 61 | W | ‘15 | F | 24 | 5/18 | 6/8 | 12 | 58 | 42 | 0 | 0 | 0 | - | Dead |
| 61.2 | CG | ‘15 | F | 23 | 6/24 | 7/15 | 21 | 43 | 57 | 0 | 0 | 0 | 2,351 | Lost |
| 64 | CG | ‘15 | F | 29 | 5/18 | 6/6 | 19 | 42 | 58 | 0 | 0 | 0 | - | Lost |
| 68 | CG | ‘15 | F | 32 | 5/18 | 7/1 | 21 | 43 | 52 | 5 | 0 | 0 | 2,351 | Captured |
| 68.2 | W | ‘15 | M | 36 | 7/10 | 7/24 | 15 | 27 | 73 | 0 | 0 | 0 | - | Lost |
| 73 | CG | ‘15 | F | 48 | 5/18 | 6/6 | 20 | 65 | 35 | 0 | 0 | 0 | 1,715 | Dead |
| 73.2 | W | ‘15 | M | 22 | 6/24 | 8/7 | 41 | 56 | 41 | 0 | 2 | 0 | 14,469 | Captured |
| 78 | CG | ‘15 | M | 49 | 5/18 | 6/26 | 37 | 32 | 59 | 8 | 0 | 0 | 3,273 | Captured |
| 78.2 | W | ‘15 | F | 45 | 7/5 | 8/10 | 36 | 0 | 36 | 53 | 11 | 0 | 13,160 | Dead |
| 80 | CG | ‘15 | M | 38 | 5/18 | 7/23 | 34 | 62 | 35 | 3 | 0 | 0 | 24,375 | Dead |
| 83 | CG | ‘15 | F | 29 | 5/18 | 5/21 | 2 | 50 | 50 | 0 | 0 | 0 | - | Dead |
| 83.2 | W | ‘15 | F | 36 | 6/24 | 8/10 | 45 | 44 | 42 | 13 | 0 | 0 | 3,135 | Dead |
| 85 | W | ‘15 | M | 29 | 5/18 | 6/20 | 29 | 21 | 69 | 7 | 3 | 0 | 21,637 | Captured |
| 85.2 | W | ‘15 | F | 26 | 6/24 | 8/8 | 44 | 7 | 93 | 0 | 0 | 0 | 3,811 | Lost |
| 0.2 | W | ‘16 | F | 48 | 5/30 | 6/15 | 15 | 20 | 80 | 0 | 0 | 0 | - | Captured |
| 0.3 | W | ‘16 | F | 35 | 6/18 | 6/22 | 5 | 80 | 20 | 0 | 0 | 0 | - | Lost |

| | | | | | | | | | | | | | | |
|---|---|---|---|---|---|---|---|---|---|---|---|---|---|---|
| 1.2 | CG | ‘16 | M | 39 | 5/30 | 6/21 | 18 | 28 | 67 | 6 | 0 | 0 | - | Lost |
| 4.2 | W | ‘16 | F | 40 | 5/30 | 7/4 | 30 | 100 | 0 | 0 | 0 | 0 | 3,756 | Dead |
| 4.3 | W | ‘16 | F | 39 | 7/15 | 7/20 | 6 | 50 | 50 | 0 | 0 | 0 | - | Dead |
| 4.4 | W | ‘16 | F | 46 | 7/15 | 8/8 | 25 | 16 | 84 | 0 | 0 | 0 | 6,218 | Captured |
| 13.2 | W | ‘16 | M | 45 | 5/30 | 7/4 | 30 | 90 | 10 | 0 | 0 | 0 | 30,535 | Dead |
| 13.3 | W | ‘16 | F | 37 | 7/15 | 8/8 | 18 | 17 | 11 | 72 | 0 | 0 | - | Captured |
| 15.3 | CG | ‘16 | F | 34 | 6/22 | 7/14 | 17 | 0 | 94 | 6 | 0 | 0 | - | Dead |
| 18 | W | ‘16 | F | 43 | 5/30 | 8/8 | 47 | 30 | 64 | 0 | 0 | 6 | 188,581 | Captured |
| 26.3 | W | ‘16 | F | 40 | 7/15 | 8/8 | 25 | 16 | 84 | 0 | 0 | 0 | 981 | Captured |
| 31.3 | CG | ‘16 | M | 33 | 5/30 | 6/15 | 15 | 40 | 60 | 0 | 0 | 0 | - | Captured |
| 31.4 | CG | ‘16 | F | 28 | 6/22 | 7/20 | 23 | 4 | 83 | 13 | 0 | 0 | 48,784 | Dead |
| 40.2 | W | ‘16 | F | 49 | 5/30 | 6/8 | 9 | 33 | 67 | 0 | 0 | 0 | - | Lost |
| 42 | W | ‘16 | M | 45 | 5/30 | 8/8 | 66 | 94 | 6 | 0 | 0 | 0 | 2,640 | Captured |
| 45 | W | ‘16 | F | 28 | 5/30 | 6/15 | 15 | 53 | 47 | 0 | 0 | 0 | - | Captured |
| 45.2 | W | ‘16 | M | 31 | 6/18 | 8/8 | 49 | 14 | 84 | 2 | 0 | 0 | 70,017 | Captured |
| 46.3 | CG | ‘16 | F | 44 | 6/6 | 6/21 | 12 | 17 | 83 | 0 | 0 | 0 | - | Lost |
| 48 | W | ‘16 | F | 32 | 5/30 | 8/8 | 39 | 8 | 72 | 21 | 0 | 0 | 91,824 | Captured |
| 50 | W | ‘16 | F | 28 | 5/30 | 6/21 | 20 | 90 | 10 | 0 | 0 | 0 | 6,423 | Lost |
| 53.2 | W | ‘16 | F | 37 | 5/30 | 6/3 | 5 | 0 | 100 | 0 | 0 | 0 | - | Lost |
| 56.2 | CG | ‘16 | M | 34 | 6/1 | 6/15 | 12 | 83 | 17 | 0 | 0 | 0 | - | Dead |
| 60 | CG | ‘16 | M | 45 | 5/30 | 6/15 | 12 | 42 | 58 | 0 | 0 | 0 | - | Dead |
| 60.2 | CG | ‘16 | F | 28 | 6/22 | 7/11 | 14 | 0 | 64 | 36 | 0 | 0 | - | Dead |

| | | | | | | | | | | | | | | |
|---|---|---|---|---|---|---|---|---|---|---|---|---|---|---|
| 60.3 | CG | ‘16 | M | 36 | 7/15 | 8/8 | 22 | 18 | 82 | 0 | 0 | 0 | 6,481 | Captured |
| 70 | CG | ‘16 | M | 31 | 5/30 | 6/2 | 4 | 0 | 100 | 0 | 0 | 0 | - | Dead |
| 73.3 | CG | ‘16 | F | 27 | 5/30 | 7/17 | 30 | 3 | 93 | 3 | 0 | 0 | 246,661 | Lost |
| 78.3 | CG | ‘16 | M | 25 | 5/30 | 8/9 | 13 | 38 | 54 | 8 | 0 | 0 | - | Captured |
| 79 | CG | ‘16 | F | 40 | 5/30 | 7/10 | 27 | 19 | 74 | 4 | 0 | 4 | 116,546 | Lost |
| 80.2 | W | ‘16 | F | 36 | 7/15 | 8/7 | 21 | 76 | 24 | 0 | 0 | 0 | 1,817 | Dead |
| 89 | CG | ‘16 | M | 24 | 5/30 | 6/28 | 24 | 83 | 17 | 0 | 0 | 0 | 3,740 | Dead |
| 89.2 | CG | ‘16 | F | 31 | 7/15 | 8/8 | 19 | 0 | 68 | 21 | 11 | 0 | - | Captured |
| 92 | CG | ‘16 | M | 30 | 5/30 | 6/26 | 18 | 0 | 67 | 28 | 0 | 6 | - | Lost |
| 94 | CG | ‘16 | F | 43 | 7/15 | 7/20 | 6 | 33 | 67 | 0 | 0 | 0 | - | Lost |

**Table S2**. Summary of 72 radio tracked northern leopard frogs in Iowa, USA 2015-2016. Sites: CG = Cerro Gordo, W = Worth; year, 2015 (’15) or 2016 (’16); mass at first capture; # of locations; proportion of locations occurring in each habitat type: W = wetland, G = grassland, A = Agriculture, F = Forest, and D = Developed; home range estimate (only individuals with ≥ 20 locations; $m^2$); and fate (dead = transmitter was found in environment, lost = could not be relocated, and captured = individual captured and euthanized for tissue analysis). ND = not detected.

| Location | ID # | Date | Atrazine | Azoxystrobin | Bifenthrin | Chlorpyrifos | Cyhalothrin | Fluxapyroxad | Metolachlor | Permethrin | Propiconazole | Pyraclostrobin | Tebuconazole | Trifloxystrobin | # of Pesticides |
|---|---|---|---|---|---|---|---|---|---|---|---|---|---|---|---|
| CG | 60.3 | 7/30/2016 | 10.7 | 21.4 | 4.80 | 10.6 | ND | 2.23 | 32.8 | ND | 7.86 | ND | 5.71 | ND | 8 |
| CG | 60.3 | 8/8/2016 | ND | 9.91 | 2.46 | 54.9 | ND | ND | ND | 9.18 | ND | ND | ND | ND | 4 |
| CG | 89.2 | 7/30/2016 | 54.4 | 16.3 | 4.24 | 8.42 | ND | ND | 3.10 | 6.76 | 5.98 | ND | 2.86 | ND | 8 |
| CG | 89.2 | 8/8/2016 | ND | 26.0 | ND | 8.82 | 2.50 | ND | ND | 5.62 | 2.30 | ND | ND | ND | 5 |
| W | 26.3 | 7/30/2016 | 6.88 | 33.0 | 8.10 | 61.0 | 7.44 | 7.22 | 3.68 | 41.9 | 5.34 | 49.3 | 5.71 | 16.9 | 12 |
| W | 26.3 | 8/8/2016 | ND | 10.9 | 11.2 | 38.4 | 3.04 | 2.60 | ND | ND | ND | ND | ND | 5.36 | 6 |
| W | 18 | 7/3/2016 | 5.76 | ND | ND | ND | ND | ND | 2.80 | ND | ND | ND | ND | ND | 2 |
| W | 18 | 7/30/2016 | ND | 21.1 | 5.60 | 128 | 7.61 | 33.2 | 6.64 | 55.4 | 4.64 | 103 | 12.9 | 6.88 | 11 |
| W | 18 | 8/8/2016 | ND | 9.10 | 5.38 | 24.7 | ND | 2.53 | ND | 6.58 | ND | ND | ND | ND | 5 |
| W | 4.4 | 7/30/2016 | 4.98 | 35.9 | 3.74 | 55.1 | 5.46 | 10.7 | 4.78 | 3.92 | 10.3 | 60.6 | 7.07 | 9.17 | 12 |
| W | 4.4 | 8/8/2016 | ND | 23.5 | 13.4 | 44.3 | 4.12 | 3.03 | ND | ND | 3.42 | ND | ND | 15.6 | 7 |
| W | 13.2 | 7/30/2016 | 23.6 | 23.9 | 5.72 | 13.7 | 2.86 | 6.89 | 56.2 | 7.86 | ND | 28.1 | ND | 5.73 | 10 |
| W | 13.2 | 8/8/2016 | ND | 15.5 | 10.9 | 25.8 | 2.86 | ND | ND | 14.6 | ND | ND | ND | 2.04 | 6 |
| W | 42 | 7/3/2016 | 11.1 | ND | ND | ND | ND | ND | ND | 7.74 | ND | ND | ND | ND | 2 |
| W | 42 | 7/17/2016 | 43.1 | 13.7 | ND | 2.98 | ND | 2.65 | 75.3 | 5.20 | 3.48 | ND | ND | 1.25 | 8 |
| W | 42 | 7/30/2016 | 23.3 | 38.9 | 8.42 | 51.0 | 5.06 | 18.2 | 28.8 | ND | 8.54 | 84.4 | 18.0 | 15.8 | 11 |
| W | 42 | 8/8/2016 | 3.24 | 20.5 | 9.26 | 29.4 | 2.38 | 3.41 | 2.48 | ND | 3.70 | 39.0 | 4.20 | 4.26 | 11 |
| W | 45.2 | 7/3/2016 | 189 | ND | ND | ND | ND | ND | 15.8 | ND | ND | ND | ND | ND | 2 |

| | | | | | | | | | | | | | | | |
|---|---|---|---|---|---|---|---|---|---|---|---|---|---|---|---|
| W | 45.2 | 7/17/2016 | ND | ND | ND | ND | ND | ND | 2.10 | 6.48 | ND | ND | ND | ND | 2 |
| W | 45.2 | 7/30/2016 | ND | 32.1 | 6.14 | 81.9 | 18.0 | 22.2 | ND | 4.18 | 9.72 | 57.7 | 3.57 | 47.5 | 10 |
| W | 45.2 | 8/8/2016 | ND | 7.78 | 7.66 | 37.0 | 2.32 | 5.49 | ND | ND | ND | 27.2 | ND | 2.85 | 7 |
| W | 48 | 7/3/2016 | ND | ND | ND | ND | ND | ND | 4.30 | ND | ND | ND | ND | ND | 1 |
| W | 48 | 7/17/2016 | ND | ND | ND | 2.34 | ND | ND | 2.48 | 3.20 | ND | ND | ND | ND | 3 |
| W | 48 | 7/30/2016 | ND | 20.7 | 4.26 | 65.1 | 5.56 | 15.0 | 9.48 | 30.1 | 3.06 | 47.7 | ND | 10.7 | 10 |
| W | 48 | 8/8/2016 | ND | ND | 7.78 | 24.6 | 2.80 | ND | ND | ND | ND | 11.7 | ND | 6.12 | 5 |

**Table S3**. Concentration of pesticides (ng/PSD) detected in silicone passive sampling devices (PSDs) deployed with radio tracked leopard frogs in Iowa, 2016. CG = Cerro Gordo, W = Worth. ND = not detected.

| Site | Habitat | Date | Atrazine | Azoxystrobin | Bifenthrin | Chlorpyrifos | Cyhalothrin | Cyproconazole | Esfenvalerate | Fluxapyroxad | Flutriafol | Imidacloprid | Metolachlor | Permethrin | Propiconazole | Pyraclostrobin | Tebuconazole | Trifloxystrobin | Trifluralin |
|---|---|---|---|---|---|---|---|---|---|---|---|---|---|---|---|---|---|---|---|
| | | | **H** | **F** | **I** | **I** | **I** | **F** | **I** | **F** | **F** | **I** | **H** | **I** | **F** | **F** | **F** | **F** | **H** |
| CG | A | 7/30/2015 | 2.40 | 3.54 | 2.22 | 5.71 | ND | ND | ND | ND | ND | ND | 3.44 | ND | 3.83 | 37.1 | 8.19 | 5.47 | ND |
| CG | A | 8/13/2015 | ND | ND | 3.54 | 116 | 12.0 | ND | 41.1 | ND | ND | ND | 2.06 | ND | 3.01 | ND | ND | ND | ND |
| CG | A | 7/3/2016 | 9.56 | ND | ND | ND | ND | ND | ND | ND | ND | ND | 504 | ND | 5.68 | ND | ND | ND | 15.7 |
| CG | A | 7/17/2016 | 6.92 | 37.7 | ND | 8.14 | ND | ND | ND | ND | ND | ND | 9.76 | ND | 19.2 | ND | ND | ND | 2.28 |
| CG | A | 7/17/2016 | 6.52 | 49.1 | ND | 6.98 | 3.66 | ND | ND | 11.8 | ND | ND | 429 | ND | 28.8 | ND | ND | ND | 7.70 |
| CG | A | 7/30/2016 | 3.70 | 28.1 | ND | 4.94 | ND | ND | ND | ND | ND | ND | ND | ND | 8.98 | ND | ND | ND | ND |
| CG | A | 7/30/2016 | 7.80 | 25.0 | 2.06 | 21.9 | ND | ND | ND | 8.84 | ND | ND | 271 | ND | 17.9 | ND | ND | ND | 5.0 |
| CG | A | 8/9/2016 | ND | 85.8 | ND | 3.72 | ND | ND | ND | 7.98 | ND | ND | ND | ND | 37.8 | ND | ND | ND | ND |
| CG | A | 8/9/2016 | ND | 234 | 3.24 | 1219 | 66.5 | ND | ND | 5.36 | ND | ND | 18.5 | ND | 287 | ND | ND | 6.66 | 3.74 |
| CG | G | 7/30/2015 | 1.90 | 13.4 | 2.38 | 3.42 | ND | 8.04 | ND | ND | 12.2 | ND | 4.16 | ND | 13.3 | 57.0 | 17.8 | 3.60 | ND |
| CG | G | 7/30/2015 | ND | 8.39 | 2.86 | 3.68 | ND | ND | ND | ND | ND | ND | 2.98 | ND | 4.78 | ND | 3.42 | ND | ND |
| CG | G | 8/13/2015 | ND | ND | ND | 28.9 | ND | 1.90 | 3.79 | ND | ND | ND | ND | ND | ND | ND | 8.52 | ND | ND |
| CG | G | 8/13/2015 | ND | ND | ND | 60.8 | ND | ND | 3.45 | ND | ND | ND | ND | ND | ND | ND | ND | ND | ND |
| CG | G | 7/3/2016 | 8.44 | ND | ND | ND | ND | ND | ND | ND | ND | ND | 5.04 | ND | ND | ND | ND | ND | ND |
| CG | G | 7/17/2016 | ND | 44.5 | ND | 3.86 | ND | ND | ND | ND | ND | ND | 4.14 | 26.8 | 14.7 | ND | ND | ND | ND |
| CG | G | 7/30/2016 | ND | 32.8 | 2.22 | 11.2 | ND | ND | ND | ND | ND | ND | ND | ND | 16.3 | ND | ND | ND | ND |

| | | | | | | | | | | | | | | | | | | | |
|---|---|---|---|---|---|---|---|---|---|---|---|---|---|---|---|---|---|---|---|
| CG | G | 8/9/2016 | ND | 25.1 | ND | 37.2 | ND | ND | ND | ND | ND | ND | ND | ND | 21.4 | ND | ND | ND | ND |
| CG | W | 7/30/2015 | 2.24 | 5.46 | ND | 4.72 | ND | 3.14 | ND | ND | 6.28 | ND | 2.36 | ND | 6.50 | 72.1 | 11.0 | 8.06 | ND |
| CG | W | 8/13/2015 | ND | 3.31 | 3.78 | 157 | ND | ND | 60.6 | ND | ND | ND | 2.48 | ND | ND | ND | ND | ND | ND |
| CG | W | 7/3/2016 | 29.4 | ND | ND | 9.12 | ND | ND | ND | ND | ND | ND | 14.6 | ND | 2.02 | ND | ND | ND | ND |
| CG | W | 8/9/2016 | 4.08 | 94.6 | ND | 141 | ND | ND | ND | 6.98 | ND | ND | 2.18 | ND | 33.9 | ND | ND | ND | ND |
| CG | W | 8/9/2016 | 8.86 | 35.3 | ND | 7.04 | ND | ND | ND | ND | ND | ND | ND | ND | 15.1 | ND | ND | ND | ND |
| W | A | 7/3/2016 | 480 | ND | ND | ND | ND | ND | ND | ND | ND | ND | 19.8 | ND | ND | ND | ND | ND | ND |
| W | A | 7/17/2016 | 62.9 | 20.3 | ND | 6.04 | ND | ND | ND | ND | ND | ND | 4.06 | ND | 6.16 | ND | ND | ND | ND |
| W | A | 7/30/2016 | 61.3 | 37.2 | 5.68 | 75.8 | 7.06 | ND | ND | 13.5 | ND | ND | 4.02 | ND | 21.2 | 66.3 | 19.3 | 27.0 | ND |
| W | A | 8/9/2016 | 15.8 | 31.2 | 14.8 | 102 | 4.08 | ND | ND | 3.40 | ND | ND | ND | ND | 11.7 | ND | ND | 14.6 | ND |
| W | G | 7/30/2015 | ND | ND | 3.00 | 4.31 | ND | ND | ND | ND | 2.73 | 5.84 | 3.04 | ND | ND | ND | ND | ND | ND |
| W | G | 7/30/2015 | 5.88 | 14.4 | 5.31 | 5.94 | ND | ND | ND | ND | ND | ND | 6.28 | ND | 19.1 | 84.9 | 17.1 | 7.40 | ND |
| W | G | 8/13/2015 | ND | ND | 6.70 | 31.4 | ND | ND | ND | ND | ND | ND | ND | ND | ND | ND | ND | ND | ND |
| W | G | 8/13/2015 | ND | ND | 4.87 | 33.0 | 1.98 | ND | ND | ND | ND | ND | ND | ND | ND | ND | ND | ND | ND |
| W | G | 7/3/2016 | 2.98 | ND | ND | ND | ND | ND | ND | ND | ND | ND | 4.09 | ND | ND | ND | ND | ND | ND |
| W | G | 7/17/2016 | 2.28 | 38.7 | ND | ND | ND | ND | ND | ND | ND | ND | 2.26 | ND | 14.8 | ND | ND | ND | ND |
| W | G | 7/30/2016 | ND | 35.1 | 2.34 | 40.2 | 7.20 | ND | ND | 26.1 | ND | ND | ND | ND | 16.2 | 80.7 | 18.40 | 24.2 | ND |
| W | G | 8/9/2016 | ND | 23.6 | 6.92 | 53.8 | 4.60 | ND | ND | ND | ND | ND | ND | ND | 16.0 | ND | ND | 24.8 | ND |
| W | W | 7/30/2015 | 7.10 | 14.3 | 2.02 | 6.62 | ND | ND | ND | ND | ND | ND | 7.64 | ND | 13.6 | 51.5 | 12.7 | 6.20 | ND |
| W | W | 7/30/2015 | 4.20 | ND | ND | 4.16 | ND | ND | ND | ND | ND | ND | 4.14 | ND | 3.52 | 15.1 | 6.06 | ND | ND |
| W | W | 8/13/2015 | ND | ND | 2.18 | 17.0 | ND | ND | ND | ND | ND | ND | ND | ND | ND | ND | ND | ND | ND |
| W | W | 8/13/2015 | ND | ND | 1.89 | 24.0 | ND | ND | ND | ND | ND | ND | ND | ND | ND | ND | 15.9 | ND | ND |

| | | | | | | | | | | | | | | | | | | | |
|---|---|---|---|---|---|---|---|---|---|---|---|---|---|---|---|---|---|---|---|
| W | W | 7/3/2016 | 120 | ND | ND | ND | ND | ND | ND | ND | ND | ND | 36.5 | ND | ND | ND | ND | ND | ND |
| W | W | 7/3/2016 | 6.38 | ND | ND | ND | ND | ND | ND | ND | ND | ND | 3.70 | ND | ND | ND | ND | ND | ND |
| W | W | 7/17/2016 | 71.5 | 30.6 | ND | ND | ND | ND | ND | 11.2 | ND | ND | 122 | ND | 19.3 | ND | ND | ND | ND |
| W | W | 7/17/2016 | 45.4 | 33.0 | ND | 5.18 | ND | ND | ND | 5.46 | ND | ND | 67.8 | ND | 17.7 | ND | ND | ND | ND |
| W | W | 7/30/2016 | 24.5 | 37.2 | ND | 5.98 | ND | ND | ND | 20.3 | ND | ND | 45.8 | ND | 15.3 | 141 | 50.3 | 8.04 | ND |
| W | W | 7/30/2016 | 38.3 | 96.4 | 3.80 | 41.9 | 3.44 | ND | ND | 44.6 | ND | ND | 154 | ND | 47.1 | 311 | 127 | 27.6 | ND |
| W | W | 8/9/2016 | 11.2 | 39.0 | ND | 41.4 | ND | ND | ND | 12.2 | ND | ND | 5.72 | ND | 14.9 | ND | 26.7 | 5.40 | ND |
| W | W | 8/9/2016 | ND | 41.8 | 5.14 | 29.0 | ND | ND | ND | 5.28 | ND | ND | ND | ND | 9.94 | ND | 12.1 | 10.7 | ND |

**Table S4.** Concentration of pesticides detected (ng/PSD) in silicone passive sampling devices (PSDs) placed from June-August in 2015 and 2016. Under pesticide names, H=Herbicide, I=Insecticide, and F=Fungicide; Site: CG = Cerro Gordo and W = Worth; Habitat: W=Wetlands, A=Agriculture, and G=Grassland; ND = not detected.

| **ID #** | **Date** | **Bifenthrin** | **Fenbuconazole** | ***p,p'*-DDE** | **Tebuconazole** |
|---|---|---|---|---|---|
| C1W | 5/24/2016 | ND | 7.30 | ND | 230 |
| C2W | 5/24/2016 | ND | 5.40 | ND | 71.8 |
| C3W | 5/24/2016 | ND | 12.6 | ND | 24.4 |
| C4W | 5/24/2016 | ND | 31.0 | ND | 23.2 |
| C5W | 5/24/2016 | ND | 41.9 | ND | 19.1 |
| C1CG | 5/24/2016 | ND | ND | 7.60 | 229 |
| C2CG | 5/24/2016 | ND | ND | ND | 34.6 |
| C3CG | 5/24/2016 | ND | 44.6 | ND | ND |
| C4CG | 5/24/2016 | ND | ND | 21.7 | 11.6 |
| C5CG | 5/24/2016 | ND | ND | 43.8 | 33.3 |
| 60.3 | 8/8/2016 | ND | ND | ND | 20.1 |
| 89.2 | 8/8/2016 | 1.40 | ND | 3.26 | 4.90 |
| 78.3 | 8/9/2016 | ND | ND | 28.0 | ND |
| 4.4 | 8/8/2016 | 2.09 | ND | 4.59 | ND |
| 13.3 | 8/8/2016 | ND | ND | ND | ND |
| 18 | 8/8/2016 | ND | ND | ND | ND |
| 26.3 | 8/8/2016 | ND | ND | ND | ND |
| 42 | 8/8/2016 | ND | ND | 21.1 | ND |

| | | | | | |
|---|---|---|---|---|---|
| 45.2 | 8/8/2016 | ND | ND | ND | ND |
| 48 | 8/8/2016 | ND | ND | ND | ND |

**Table S5**. Pesticides detected (ng/g) in tissues of captured and euthanized northern leopard frogs in Iowa. ND = not detected.

| | | | | | Surrounding land use (%) | | | |
|---|---|---|---|---|---|---|---|---|
| **Study Site Location** | **Construction Year** | **Wetland (ha)** | **Grass Buffer (ha)** | **Drainage (ha)** | **Agriculture** | **Grassland** | **Wetland** | **Other** |
| Cerro Gordo | 2006 | 1.5 | 6.0 | 298 | 77.7 | 9.0 | 1.5 | 12.0 |
| Worth | 2008 | 2.4 | 9.8 | 287 | 51.7 | 22.1 | 16.8 | 9.4 |

**Table S6**. Study site information at the two study locations. Ha= hectares; Other = human developed or forest. Land-use was measured using a 1 km buffer around each wetland.

| ID # | CG F 15 | CG M 15 | CG F 16 | CG M 16 | W F 15 | W M 15 | W F 16 | W M 16 |
|---|---|---|---|---|---|---|---|---|
| 0 | 0 | 1 | 0 | 0 | 0 | 0 | 0 | 0 |
| 0.2 | 0 | 0 | 0 | 0 | 0 | 0 | 1 | 0 |
| 0.3 | 0 | 0 | 0 | 0 | 0 | 0 | 1 | 0 |
| 1 | 0 | 0 | 0 | 0 | 1 | 0 | 0 | 0 |
| 1.2 | 0 | 0 | 0 | 1 | 0 | 0 | 0 | 0 |
| 4 | 0 | 1 | 0 | 0 | 0 | 0 | 0 | 0 |
| 4.2 | 0 | 0 | 0 | 0 | 0 | 0 | 1 | 0 |
| 4.3 | 0 | 0 | 0 | 0 | 0 | 0 | 1 | 0 |
| 4.4 | 0 | 0 | 0 | 0 | 0 | 0 | 1 | 0 |
| 6 | 0 | 0 | 0 | 0 | 1 | 0 | 0 | 0 |
| 6.2 | 0 | 1 | 0 | 0 | 0 | 0 | 0 | 0 |
| 13 | 0 | 1 | 0 | 0 | 0 | 0 | 0 | 0 |
| 13.2 | 0 | 0 | 0 | 0 | 0 | 0 | 0 | 1 |
| 13.3 | 0 | 0 | 0 | 0 | 0 | 0 | 1 | 0 |
| 15 | 0 | 0 | 0 | 0 | 0 | 1 | 0 | 0 |
| 15.2 | 0 | 0 | 0 | 0 | 0 | 1 | 0 | 0 |
| 15.3 | 0 | 0 | 1 | 0 | 0 | 0 | 0 | 0 |
| 18 | 0 | 0 | 0 | 0 | 0 | 0 | 1 | 0 |
| 23 | 0 | 0 | 0 | 0 | 0 | 1 | 0 | 0 |
| 26 | 0 | 0 | 0 | 0 | 0 | 1 | 0 | 0 |
| 26.2 | 1 | 0 | 0 | 0 | 0 | 0 | 0 | 0 |

| | | | | | | | | |
|---|---|---|---|---|---|---|---|---|
| 26.3 | 0 | 0 | 0 | 0 | 0 | 0 | 1 | 0 |
| 29 | 1 | 0 | 0 | 0 | 0 | 0 | 0 | 0 |
| 29.2 | 0 | 1 | 0 | 0 | 0 | 0 | 0 | 0 |
| 31 | 1 | 0 | 0 | 0 | 0 | 0 | 0 | 0 |
| 31.2 | 0 | 0 | 0 | 0 | 1 | 0 | 0 | 0 |
| 31.3 | 0 | 0 | 0 | 1 | 0 | 0 | 0 | 0 |
| 31.4 | 0 | 0 | 1 | 0 | 0 | 0 | 0 | 0 |
| 34 | 1 | 0 | 0 | 0 | 0 | 0 | 0 | 0 |
| 34.2 | 1 | 0 | 0 | 0 | 0 | 0 | 0 | 0 |
| 40 | 0 | 0 | 0 | 0 | 0 | 1 | 0 | 0 |
| 40.2 | 0 | 0 | 0 | 0 | 0 | 0 | 1 | 0 |
| 42 | 0 | 0 | 0 | 0 | 0 | 0 | 0 | 1 |
| 45 | 0 | 0 | 0 | 0 | 0 | 0 | 1 | 0 |
| 45.2 | 0 | 0 | 0 | 0 | 0 | 0 | 0 | 1 |
| 46 | 0 | 0 | 0 | 0 | 0 | 1 | 0 | 0 |
| 46.2 | 1 | 0 | 0 | 0 | 0 | 0 | 0 | 0 |
| 46.3 | 0 | 0 | 1 | 0 | 0 | 0 | 0 | 0 |
| 48 | 0 | 0 | 0 | 0 | 0 | 0 | 1 | 0 |
| 49 | 0 | 0 | 0 | 0 | 0 | 1 | 0 | 0 |
| 50 | 0 | 0 | 0 | 0 | 0 | 0 | 1 | 0 |
| 53 | 0 | 1 | 0 | 0 | 0 | 0 | 0 | 0 |
| 53.2 | 0 | 0 | 0 | 0 | 0 | 0 | 1 | 0 |

| | | | | | | | | |
|---|---|---|---|---|---|---|---|---|
| 56 | 0 | 0 | 0 | 0 | 0 | 1 | 0 | 0 |
| 56.2 | 0 | 0 | 0 | 1 | 0 | 0 | 0 | 0 |
| 59 | 0 | 0 | 0 | 0 | 1 | 0 | 0 | 0 |
| 60 | 0 | 0 | 0 | 1 | 0 | 0 | 0 | 0 |
| 60.2 | 0 | 0 | 1 | 0 | 0 | 0 | 0 | 0 |
| 60.3 | 0 | 0 | 0 | 1 | 0 | 0 | 0 | 0 |
| 61 | 0 | 0 | 0 | 0 | 1 | 0 | 0 | 0 |
| 61.2 | 1 | 0 | 0 | 0 | 0 | 0 | 0 | 0 |
| 64 | 1 | 0 | 0 | 0 | 0 | 0 | 0 | 0 |
| 68 | 1 | 0 | 0 | 0 | 0 | 0 | 0 | 0 |
| 68.2 | 0 | 0 | 0 | 0 | 0 | 1 | 0 | 0 |
| 70 | 0 | 0 | 0 | 1 | 0 | 0 | 0 | 0 |
| 73 | 1 | 0 | 0 | 0 | 0 | 0 | 0 | 0 |
| 73.2 | 0 | 0 | 0 | 0 | 0 | 1 | 0 | 0 |
| 73.3 | 0 | 0 | 1 | 0 | 0 | 0 | 0 | 0 |
| 78 | 0 | 1 | 0 | 0 | 0 | 0 | 0 | 0 |
| 78.2 | 0 | 0 | 0 | 0 | 1 | 0 | 0 | 0 |
| 78.3 | 0 | 0 | 0 | 1 | 0 | 0 | 0 | 0 |
| 79 | 0 | 0 | 1 | 0 | 0 | 0 | 0 | 0 |
| 80 | 0 | 1 | 0 | 0 | 0 | 0 | 0 | 0 |
| 80.2 | 0 | 0 | 0 | 0 | 0 | 0 | 1 | 0 |
| 83 | 1 | 0 | 0 | 0 | 0 | 0 | 0 | 0 |

| | | | | | | | | |
|---|---|---|---|---|---|---|---|---|
| 83.2 | 0 | 0 | 0 | 0 | 1 | 0 | 0 | 0 |
| 85 | 0 | 0 | 0 | 0 | 0 | 1 | 0 | 0 |
| 85.2 | 0 | 0 | 0 | 0 | 1 | 0 | 0 | 0 |
| 89 | 0 | 0 | 0 | 1 | 0 | 0 | 0 | 0 |
| 89.2 | 0 | 0 | 1 | 0 | 0 | 0 | 0 | 0 |
| 92 | 0 | 0 | 0 | 1 | 0 | 0 | 0 | 0 |
| 94 | 0 | 0 | 1 | 0 | 0 | 0 | 0 | 0 |
| **Total** | **11** | **8** | **8** | **9** | **8** | **11** | **14** | **3** |

**Table S7**. Groups individual adult leopard frogs were assigned to for known fate analysis. Groups are organized by Site Sex Year. CG = Cerro Gordo, W = Worth, F=Female, M=Male, 15 = 2015, and 16 = 2016. "1" indicates frogs were in that group, with group totals in the last row.

| Model Name | k | Model Description |
|---|---|---|
| S(.) | 1 | Survival is constant for all individuals |
| S(t) | 86 | Survival varies over time |
| S(Sex) | 2 | Survival varies by sex |
| S(Year) | 2 | Survival varies by study year |
| S(Site) | 2 | Survival varies by site |
| S(Group) | 8 | Survival varies by group |
| S(.+Habitat) | 2 | Survival is constant with an additive habitat effect |
| S(t+Habitat) | 87 | Survival varies over time with an additive habitat effect |
| S(Sex+Habitat) | 3 | Survival varies by sex with an additive habitat effect |
| S(Year+Habitat) | 3 | Survival varies by study year with an additive habitat effect |
| S(Site+Habitat) | 3 | Survival varies by site with an additive habitat effect |
| S(Group+Habitat) | 9 | Survival varies by group with an additive habitat effect |

**Table S8**. Known fate models fit to data from radio telemetered leopard frogs in Iowa, k = number of parameters, S = survival, "." = constant survival, t=time, Group = individual group assignment (see Appendix III), Habitat= Grassland, Wetland, Agriculture, Forest, or Developed, Year = 2015 or 2016.

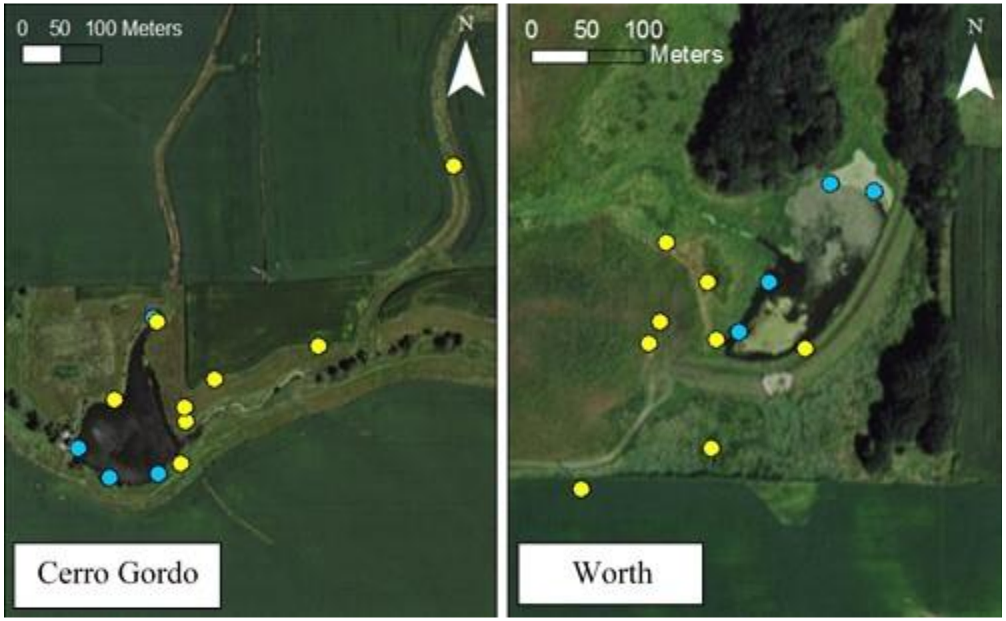


**Figure S1.** Locations of PSDs in wetlands in 2015 and 2016. Blue dots represent PSDs placed overwinter while yellow dots represent PSDs placed in different habitats during the summer. This map was created using ArcGIS 10.3.1 software, http://desktop.arcgis.com/en/arcmap/

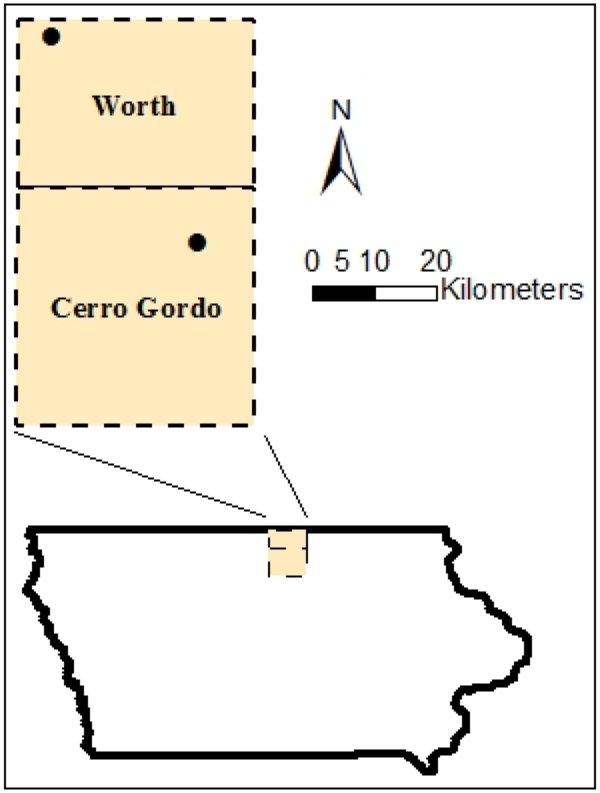


**Figure S2**. Location of two northern leopard frog radio telemetry study sites in Cerro Gordo and Worth counties, Iowa, USA. This map was created using ArcGIS 10.3.1 software, http://desktop.arcgis.com/en/arcmap/

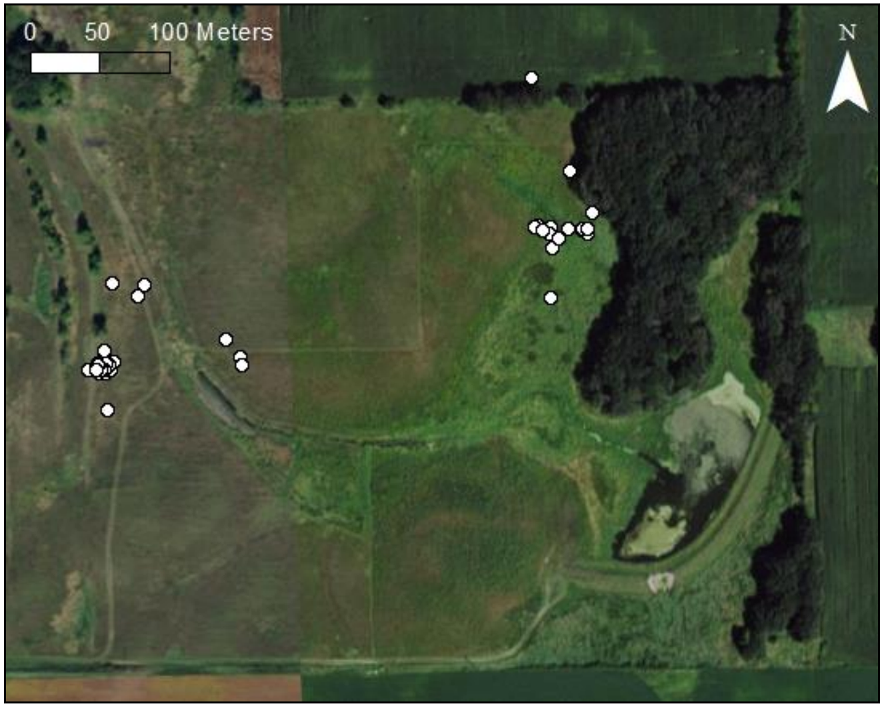


**Figure S3**. White dots illustrate locations of a radio tracked frog (individual 45.2) and placement of PSD, 18 Jun – 8 Aug, 2016 (Worth County). This map was created using ArcGIS 10.3.1 software, http://desktop.arcgis.com/en/arcmap/.

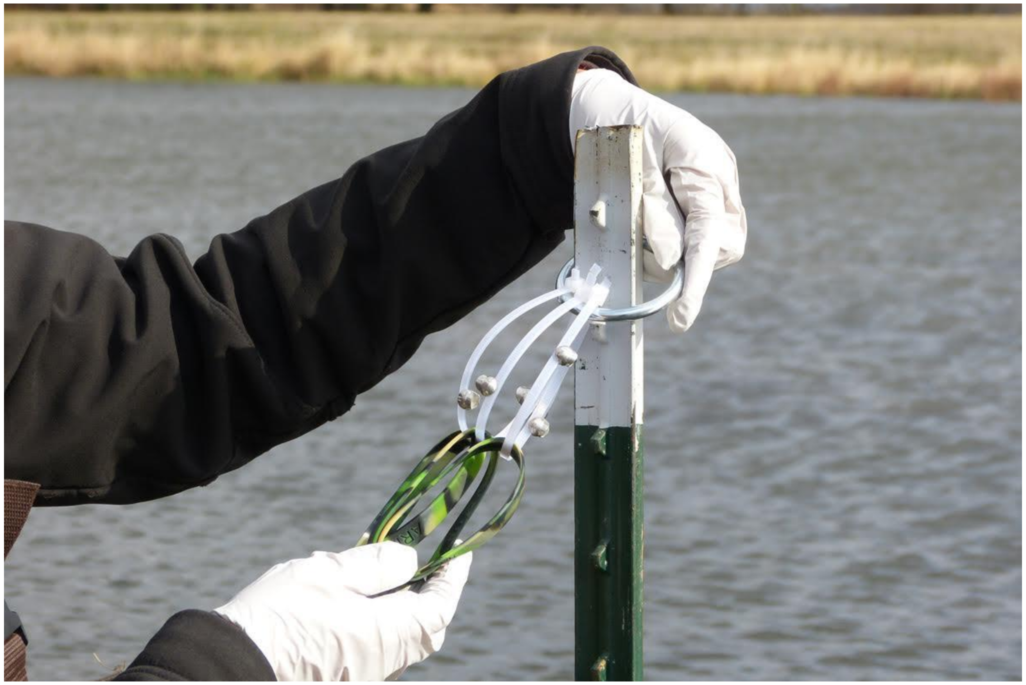

**Figure S4**. PSDs being placed in wetlands to assess pesticide exposure of northern leopard frogs during aquatic hibernation from November 2015 – April 2016 in Iowa.
